# BRG1 Links TLR4 Trans-Activation to LPS-Induced SREBP1a Expression and Liver Injury

**DOI:** 10.3389/fcell.2021.617073

**Published:** 2021-03-18

**Authors:** Wenhui Dong, Yuwen Zhu, Yangxi Zhang, Zhiwen Fan, Ziyu Zhang, Xiangshan Fan, Yong Xu

**Affiliations:** ^1^Key Laboratory of Targeted Invention of Cardiovascular Disease and Collaborative Innovation Center for Cardiovascular Translational Medicine, Department of Pathophysiology, Nanjing Medical University, Nanjing, China; ^2^Department of Pathology, Affiliated Nanjing Drum Tower Hospital of Nanjing University School of Medicine, Nanjing, China; ^3^Key Laboratory of Women’s Reproductive Health of Jiangxi, Jiangxi Provincial Maternal and Child Health Hospital, Nanchang, China; ^4^Central Laboratory, Jiangxi Provincial Maternal and Child Health Hospital, Nanchang, China; ^5^Institute of Biomedical Research, Liaocheng University, Liaocheng, China

**Keywords:** liver injury, LPS, septic shock, transcriptional regulation, inflammation, apoptosis

## Abstract

Multiple organ failure is one of the most severe consequences in patients with septic shock. Liver injury is frequently observed during this pathophysiological process. In the present study we investigated the contribution of Brahma related gene 1 (BRG1), a chromatin remodeling protein, to septic shock induced liver injury. When wild type (WT) and liver conditional BRG1 knockout (LKO) mice were injected with lipopolysaccharide (LPS), liver injury was appreciably attenuated in the LKO mice compared to the WT mice as evidenced by plasma ALT/AST levels, hepatic inflammation and apoptosis. Of interest, there was a down-regulation of sterol response element binding protein 1a (SREBP1a), known to promote liver injury, in the LKO livers compared to the WT livers. BRG1 did not directly bind to the SREBP1a promoter. Instead, BRG1 was recruited to the toll-like receptor 4 (TLR4) promoter and activated TLR4 transcription. Ectopic TLR4 restored SREBP1a expression in BRG1-null hepatocytes. Congruently, adenovirus carrying TLR4 or SREBP1a expression vector normalized liver injury in BRG1 LKO mice injected with LPS. Finally, a positive correlation between BRG1 and TLR4 expression was detected in human liver biopsy specimens. In conclusion, our data demonstrate that a BRG1-TLR4-SREBP1a axis that mediates LPS-induced liver injury in mice.

## Introduction

Septic shock, or septicemia, is a common pathophysiological process taking place in a wide range of occasions including infection, trauma, diabetes, and cirrhosis (Hotchkiss et al., 2016). Each year, incidents of septic shock trigger over 3,000,000 emergency visits and are associated with a ∼10% mortality rate in the United States alone (Wang et al., 2017). The most severe consequence of septic shock is the simultaneous dysfunction or failure of multiple organs (Azevedo et al., 2008). Liver dysfunction is common in patients with septic shock, which can be generally characterized as elevated plasma levels of alanine transaminase, alkaline phosphatase, and bilirubin (jaundice). Although liver failure is rare, liver injury significantly exacerbates the prognosis in patients with septic shock (Waseem et al., 2018).

The pathogenesis of liver dysfunction following septic shock is complex and not entirely clear at present. It is proposed that massive death of hepatocytes due to insufficient hepatic perfusion secondary to hypotension may be one of the major causes of sepsis-associated liver injury (Hotchkiss et al., 1999). Indeed, deletion of pro-apoptotic molecules such as JNK2 (Wang et al., 2006), perforin (Kuhla et al., 2009), and TNFR (Nowak et al., 2000), blocks apoptosis of hepatocytes and attenuates liver injury in mouse models of sepsis. Alternatively, altered inflammatory response is considered another critical contributing factor to sepsis-induced liver injury. It has been well-documented that enteric microbes and their components [e.g., lipopolysaccharide (LPS)] trans-located to the liver due to the disruption of the intestinal barrier function, combined with hepatic ischemia, illicit hepatic inflammation during sepsis (Yan and Li, 2014). A host of immune cells can be detected to infiltrate the liver following septic shock, including macrophages (Guillot and Tacke, 2019), T lymphocytes (Wesche-Soldato et al., 2007), myeloid-derived suppressor cells (Sander et al., 2010), and dentritic cells (Fan et al., 2015). It is generally agreed that excessively strong pro-inflammatory response precipitates liver injury in septic shock. For instance, depletion of the pro-inflammatory Kupffer cells by clodronate (Traeger et al., 2010) or NK cells by injection of anti-asialo GM1 antibodies suppresses hepatic inflammation and promotes survival in the septic mice. In addition, hepatocyte-specific deletion or pharmaceutical inhibition of toll-like receptor 4 (TLR4), a master regulator of cellular inflammation to which LPS binds, attenuates liver injury following sepsis (Deng et al., 2013; Engelmann et al., 2020). How TLR4 expression is regulated in this process is not well understood.

Brahma related gene 1 (BRG1), is an epigenetic regulator of gene expression by functioning as the catalytic component of the mammalian SWI/SNF chromatin remodeling complex. BRG1 is universally expressed in various tissues and cells and is essential for organogenesis in mice (Bultman et al., 2000). BRG1 is dispensable for normal liver function in mice under physiological conditions (Li et al., 2018a, b). Recently, we have made several discoveries that portray BRG1 as a critical modulator of liver pathologies: mice with a selective deficiency of BRG1 in hepatocytes are protected from acetaminophen-induced acute liver failure and diet induced steatosis (Kong et al., 2018; Li et al., 2018a, b, 2019b, c; Fan et al., 2019, 2020; Liu et al., 2019a). Here we report that the BRG1 conditional knockout liver conditional BRG1 knockout (LKO) mice display an ameliorated phenotype of liver injury induced by septic shock. Mechanistically, BRG1 directly binds to the TLR4 promoter to activate TLR4 transcription leading to increased expression of SREBP1a, a key regulator of inflammation and apoptosis. Therefore, our data reinforce the notion that targeting BRG1 bring may be associated with benefits in liver injury.

## Materials and Methods

### Animals

All animal experiments were reviewed and approved by the intramural Ethics Committee on Humane Treatment of Experimental Animals. *Smarca4*^*f/f*^ mice (Dong et al., 2020) were crossed to *Alb*-Cre mice (Fan et al., 2020) to generate LKO mice. The mice were maintained under the SPF environment with 12 h light/dark cycles and libitum access to food and water. Liver injury was induced in male, 8-week old BRG1 conditional knockout (LKO) mice and wild type (WT) littermates by peritoneal injection of a single dose of LPS (15 mg/kg, Sigma) as previously described (Liu et al., 2018). In certain experiments, the animals were injected via tail vein adenovirus carrying expression vectors for TLR4 or SREBP1a.

### Histology

Histological analyses were performed essentially as described before (Li et al., 2019a). Briefly, the paraffin embedded sections were blocked with 10% normal goat serum for 1 h at room temperature and then incubated with an anti-F4/80 (Proteintech, 28463-1) antibody. Staining was visualized by incubation with anti-rabbit secondary antibody and developed with a streptavidin-horseradish peroxidase kit (Pierce) for 20min. Pictures were taken using an Olympus IX-70 microscope.

### Terminal Deoxynucleotidyltransferase dUTP Nick End Labeling Assay

Terminal Deoxynucleotidyltransferase dUTP Nick End Labeling (TUNEL) assay was performed as previously described (Liu et al., 2019b; Zhao et al., 2019). Briefly, paraffin sections were incubated in the TUNEL reaction mixture (R&D Systems, 4810-30-K) at 37°C for 60 min. After several rinses with PBS, the sections were incubated with an anti-HRP antibody at room temperature for 30min. Pictures were taken using an Olympus IX-70 microscope.

### Cell Culture, Plasmids, and Transient Transfection

Human hepatoma cells (HepG2) were maintained in DMEM supplemented with 10% fetal bovine serum (FBS, Hyclone). Primary hepatocytes were isolated and cultured as previously described (Fan et al., 2019). Small interfering RNAs targeting BRG1 are: #1, AACATGCACCAGATGCACAAG and #2, GCCCATGGAGTCCATGCAT. Transient transfections were performed with Lipofectamine 2000 (Invitrogen). 24 h after transfection, LPS (1 mg/ml) was added and incubated with the cells for additional 12 h. Experiments were routinely performed in triplicate wells and repeated at least three times.

### Protein Extraction and Western Blot

Whole cell lysates were obtained by re-suspending cell pellets in RIPA buffer (50 mM Tris pH7.4, 150 mMNaCl, 1% Triton X-100) with freshly added protease inhibitor (Roche) as previously described (Lu et al., 2019; Yang et al., 2019a, b; Mao et al., 2020). Western blot analyses were performed with anti-BRG1 (Santa Cruz, sc-10768), anti-SREBP1 (Proteintech, 14088-1), anti-TLR4 (Proteintech, 19811-1), and anti-β-actin (Sigma, A2228) antibodies.

### RNA Isolation and Real-Time PCR

RNA was extracted with the RNeasy RNA isolation kit (Qiagen). Reverse transcriptase reactions were performed using a SuperScript First-strand Synthesis System (Invitrogen) as previously described (Liu et al., 2019b; Zhao et al., 2019). Real-time PCR reactions were performed on an ABI Prism 7,500 system with the following primers: human *TLR4*, 5′-CCCTGAGGCATTTAGGCAGCTA-3′ and 5′-AGGTAGAGAGGTGGCTTAGGCT-3′; human *BRG1*, 5′- TCATGTTGGCGAGCTATTTCC-3′ and 5′-GGTTCCGAAGT CTCAACGATG-3′; human *SREBP1a*, 5′-CGGCGCTGCTG ACCGACATC-3′ and 5′-CCCTGCCCCACTCCCAGCAT-3′; mouse *Tlr4*, 5′-CAAGGGATAAGAACGCTGAGA-3′ and 5′-GCAATGTCTCTGGCAGGTGTA-3′; mouse *Srebp1a*, 5′-ATGGACGAGCTGGCCTTCGGTGAGGCGGC-3′ and 5′-CA GGAAGGCTTCCAGAGAGGA-3′; mouse *Il1b*, 5′-TGGACCT TCCAGGATGAGGACA-3′ and 5′-GTTCATCTCGGAGCC TGTAGTG-3′; mouse *Il6*, 5′-TACCACTTCACAAGTCGGA GGC-3′ and 5′-CTGCAAGTGCATCATCGTTGTTC-3′; mouse *Tnfa*, 5′-GGTGCCTATGTCTCAGCCTCT-3′ and 5′-CATC GGCTGGCACCACTAGTT-3′; mouse *Bax*, 5′-CGGCGAA TTGGAGATGAACTG-3′ and 5′-GCAAAGTAGAAGAGGG CAACC-3′; mouse *Bim*, 5′-CGACAGTCTCAGGAGGAACC-3′ and 5′-CCTTCTCCATACCAGACGGA-3′; mouse *Noxa*, 5′-TCAGGAAGATCGGAGACAAA-3′ and 5′-TGAGCA CACTCGTCCTTCAA-3′. Ct values of target genes were normalized to the Ct values of housekeekping control gene (18s, 5′-CGCGGTTCTATTTTGTTGGT-3′ and 5′-TCGTCTTCGAAACTCCGACT-3′ for both human and mouse genes) using the ΔΔCt method and expressed as relative mRNA expression levels compared to the control group which is arbitrarily set as 1.

### Chromatin Immunoprecipitation

Chromatin immunoprecipitation (ChIP) assays were performed essentially as described before (Chen et al., 2020a, b, c; Dong et al., 2020; Fan et al., 2020; Li et al., 2020a, b, c; Lv et al., 2020; Mao et al., 2020; Sun et al., 2020; Wu et al., 2020; Yang et al., 2020). In brief, chromatin in control and treated cells were cross-linked with 1% formaldehyde. Cells were incubated in lysis buffer (150 mMNaCl, 25 mM Tris pH 7.5, 1% Triton X-100, 0.1% SDS, 0.5% deoxycholate) supplemented with protease inhibitor tablet and PMSF. DNA was fragmented into ∼200 bp pieces using a Branson 250 sonicator. Aliquots of lysates containing 200 μg of protein were used for each immunoprecipitation reaction with anti-BRG1 (Santa Cruz, sc-10768), anti-NF-κB (Santa Cruz, sc-372), anti-Sp1 (Abcam, ab227383), or pre-immune IgG.

### Human Specimen Collection

Liver biopsies were collected from patients with acute liver injury (ALI) referring to Nanjing Drum Tower Hospital. Control liver samples were collected from donors whose livers were deemed unsuitable for transplantation. Written informed consent was obtained from subjects or families of liver donors. All procedures that involved human samples were approved by the Ethics Committee of Nanjing Drum Tower Hospital and adhered to the principles outlined in the Declaration of Helsinki.

### Statistical Analysis

One-way ANOVA with *post hoc* Scheffe analyses were performed by SPSS software (IBM SPSS v18.0, Chicago, IL, United States). Unless otherwise specified, values of *p* < 0.05 were considered statistically significant.

## Results

### BRG1 Deficiency in Hepatocyte Alleviates LPS-Induced Liver Injury

**FIGURE 1:**
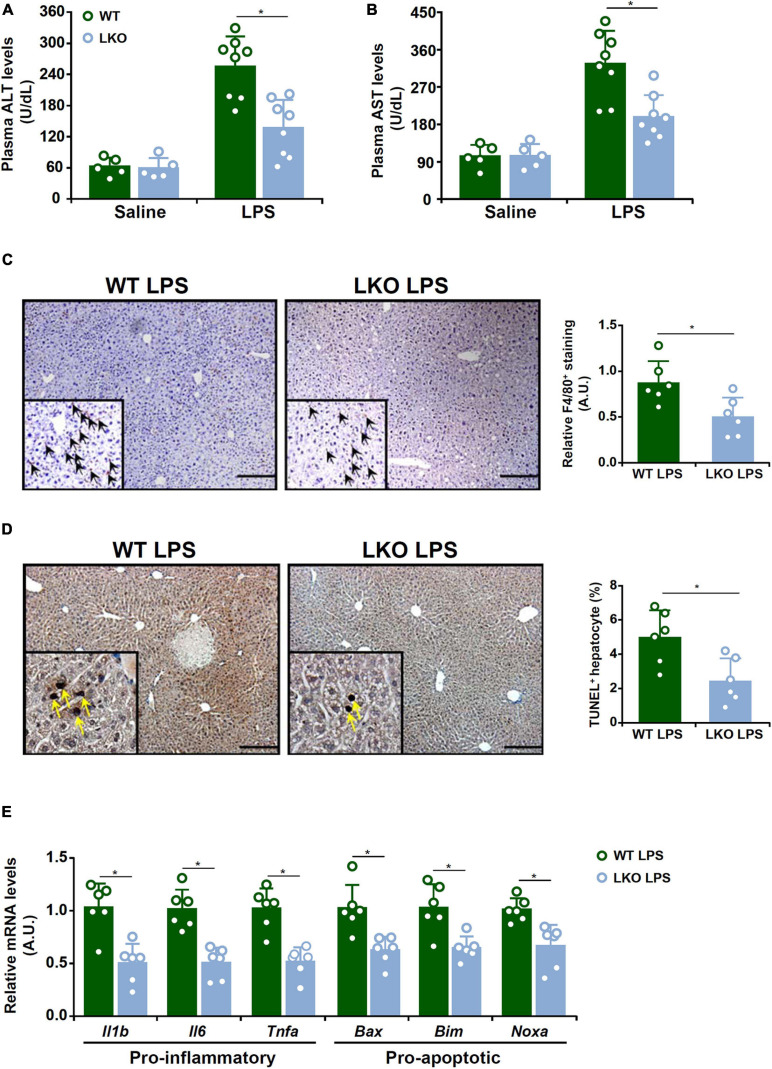
BRG1 deficiency in hepatocyte alleviates LPS-induced liver injury. WT and BRG1 LKO mice were injected peritoneally with LPS (15 mg/kg) and sacrificed 24 h after the injection. **(A)** Plasma ALT levels. *N* = 5 mice for the saline groups and *N* = 8 mice for the LPS groups. **(B)** Plasma AST levels. *N* = 5 mice for the saline groups and *N* = 8 mice for the LPS groups. **(C)** Paraffin sections were stained with F4/80. *N* = 6 mice for each group. **(D)** Paraffin sections were stained with TUNEL. *N* = 6 mice for each group. **(E)** Gene expression levels were examined by qPCR. *N* = 6 mice for each group.

We first evaluated the effect of BRG1 deletion in hepatocyte on LPS-induced liver injury. To this end, the BRG1 LKO mice and the WT mice were injected peritoneally with LPS and sacrificed 24 h after the injection. Quantification of plasma ALT levels (Figure 1A) and plasma AST levels (Figure 1B) showed that liver injury was attenuated in the LKO mice compared to the WT mice. Immunohistochemical staining with an anti-F4/80 antibody showed that there were fewer infiltrated macrophages in the LKO livers than in the WT livers (Figure 1C). The LKO mice also exhibited reduced apoptosis of hepatocytes as assessed by TUNEL staining (Figure 1D). Quantitative PCR results showed that the expression levels of several pro-inflammatory mediators, including *Il-1b*, *Il-6*, and *Tnf-a*, as well as several pro-apoptotic molecules, including *Bax*, *Bim*, and *Noxa*, were down-regulated in the LKO livers compared to the WT livers (Figure 1E).

### BRG1 Deficiency Attenuates LPS-Induced SREBP1a Expression *in vivo* and *in vitro*

**FIGURE 2:**
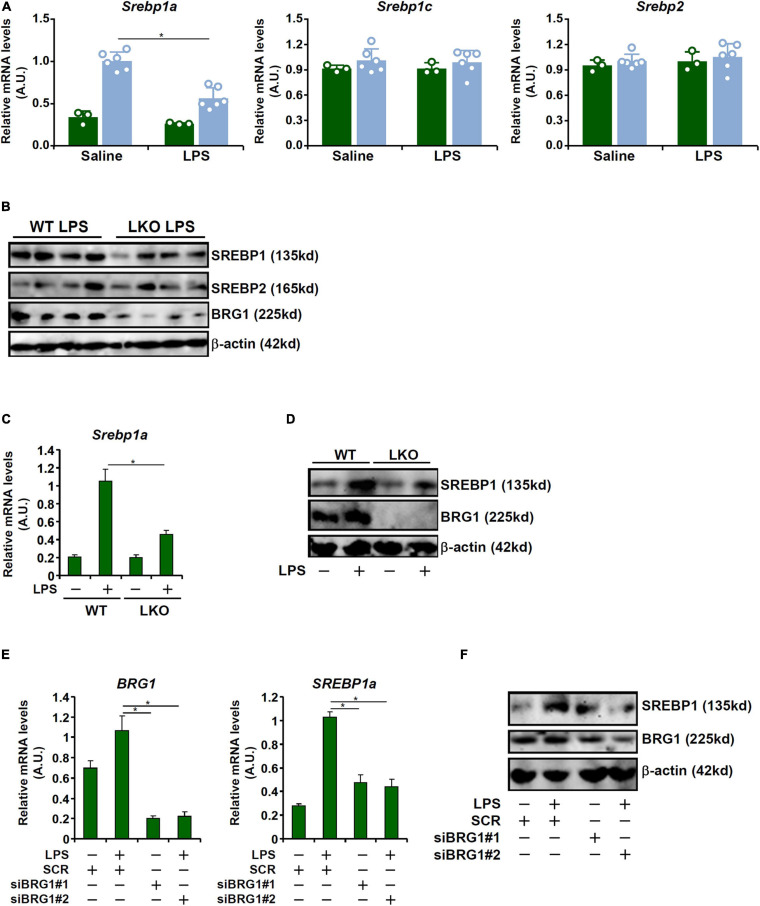
BRG1 deficiency attenuates LPS-induced SREBP1a expression *in vivo* and *in vitro*. **(A,B)** WT and BRG1 LKO mice were injected peritoneally with LPS (15 mg/kg) and sacrificed 24 h after the injection. SREBP expression levels were examined by qPCR and Western. *N* = 3 mice for the saline groups and *N* = 6 mice for the LPS groups. **(C,D)** Primary hepatocytes isolated from WT and BRG1 LKO mice were exposed to LPS (1 μg/ml) for 12 h and SREBP1a expression was examined by qPCR and Western. **(E,F)** HepG2 cells were transfected with siRNA targeting BRG1 or scrambled siRNA (SCR) followed by treatment with LPS (1 μg/ml) for 12 h and SREBP1a expression was examined by qPCR and Western.

Recent investigations have implicates SREBP family of proteins in the regulation of LPS-induced inflammatory response (Im et al., 2011; Lee et al., 2018). We hypothesized that BRG1 may contribute to LPS induced liver injury through modulating SREBP expression levels. As shown in Figures 2A,B, out of the three SREBP isoforms, SREBP1a levels were up-regulated in the liver following LPS injection whereas BRG1 deficiency dampened the induction of SREBP1a. Neither SREBP1c nor SREBP2 was influenced by LPS injection or BRG1 deficiency. In cultured hepatocytes (HepG2), BRG1 knockdown by two different pairs of siRNAs suppressed the induction of SREBP1a by LPS treatment at mRNA (Figure 2C) and protein (Figure 2D) levels. In addition, primary hepatocytes isolated from the WT mice responded better to LPS treatment than those from the BRG1 LKO mice by activating more SREBP1a molecules (Figures 2E,F).

### BRG1 Contributes to SREBP1a Induction by LPS via TLR4

**FIGURE 3:**
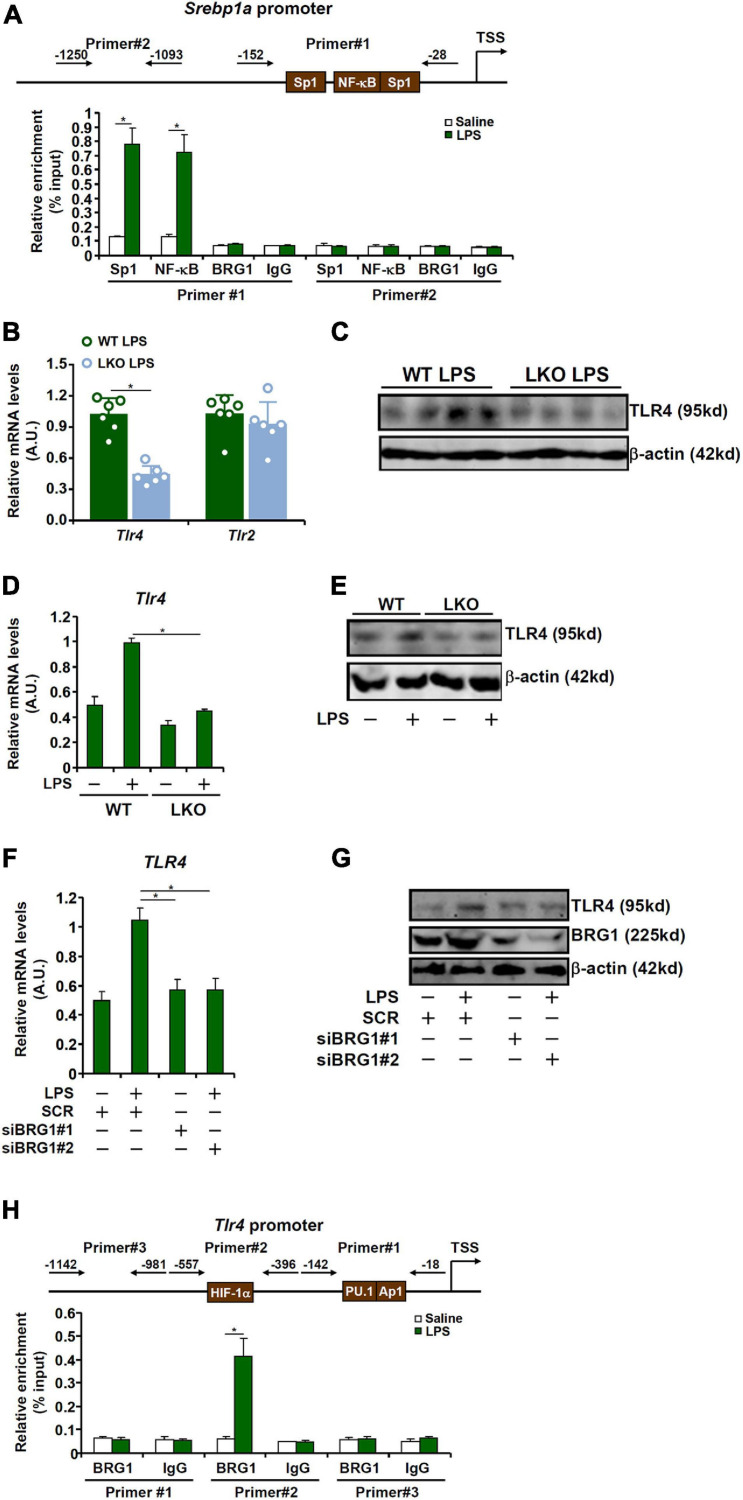
BRG1 contributes to SREBP1a induction by LPS via TLR4. **(A)** Primary murine hepatocytes were treated with or without LPS (1 μg/ml) for 12 h. ChIP assays were performed with anti-Sp1, anti-NF-κB, anti-BRG1, or IgG. **(B,C)** WT and BRG1 LKO mice were injected peritoneally with LPS (15 mg/kg) and sacrificed 24 h after the injection. TLR expression level was examined by qPCR and Western. **(D,E)** Primary hepatocytes isolated from WT and BRG1 LKO mice were exposed to LPS (1 μg/ml) for 12 h and TLR4 expression was examined by qPCR and Western. **(F,G)** HepG2 cells were transfected with siRNA targeting BRG1 or scrambled siRNA (SCR) followed by treatment with LPS (1 μg/ml) for 12 h and TLR4 expression was examined by qPCR and Western. **(H)** Primary murine hepatocytes were treated with or without LPS (1 μg/ml) for 12 h. ChIP assays were performed with anti-BRG1 or IgG.

Previous studies have identified the presence of several conserved motifs for sequence-specific transcription factors, including Sp1 and NF-κB (Zhang et al., 2005), on the proximal SREBP1a promoter (Figure 3A, upper panel). ChIP assay confirmed that in response to LPS stimulation, both Sp1 and NF-kB were recruited to the proximal, but not the distal, SREBP1a promoter (Figure 3A). Of interest, although BRG1 has been shown to interact with Sp1 (Li et al., 2019c) and NF-κB (Fang et al., 2013), no discernable BRG1 binding was detected on either the proximal or the distal SREBP1a promoter (Figure 3A), indicating that BRG1 may contribute to SREBP1a transcription indirectly.

LPS induced pro-inflammatory and pro-apoptotic signaling can be mediated through the TLR4 receptor (Park and Lee, 2013) and the TLR2 receptor (Good et al., 2012). QPCR (Figure 3B) and Western blotting (Figure 3C) showed that BRG1 deficiency down-regulated TLR4 expression but not TLR2 expression. Indeed, LPS induced TLR4 expression in primary hepatocytes isolated from the WT mice but not the BRG1 LKO mice (Figures 3D,E). Further, BRG1 depletion dampened TLR4 induction by LPS treatment in HepG2 cells (Figures 3F,G). We then hypothesized that BRG1 might directly bind to the TLR4 promoter to activate TLR4 transcription. ChIP assay (Figure 3H) showed that LPS treatment promoted BRG1 recruitment to a region of the TLR4 promoter that contains a conserved HIF-1α site, but not to a more proximal region of the TLR4 promoter that contains both a PU.1 site and an AP-1 site or to the more distal TLR4 promoter.

### TLR4 Over-Expression Rescues SREBP1a Induction by LPS in Hepatocytes

**FIGURE 4:**
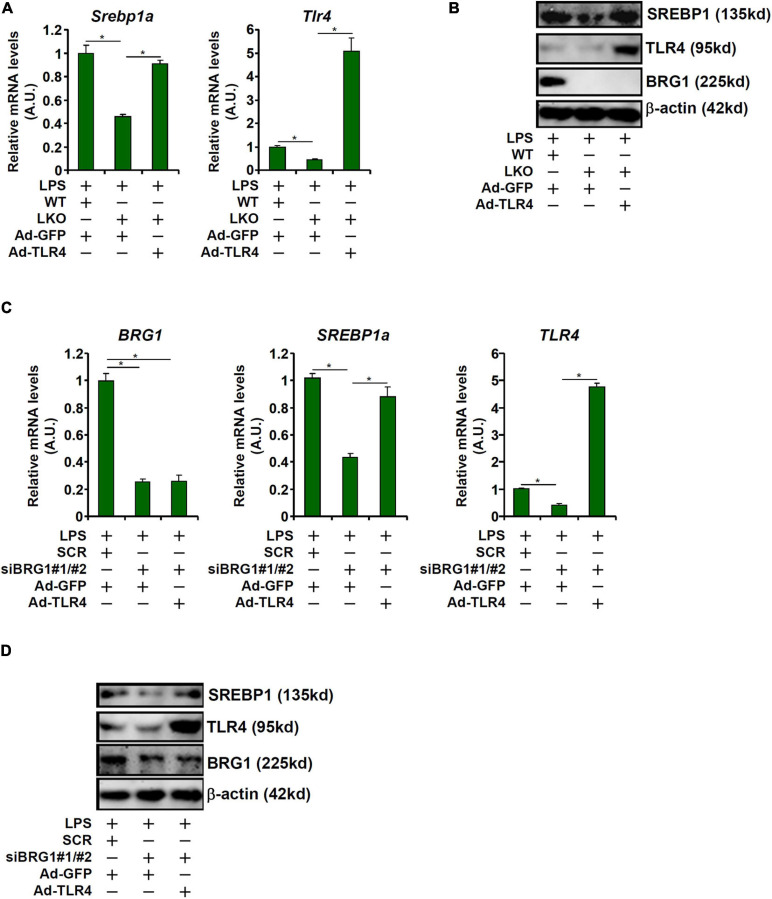
TLR4 over-expression rescues SREBP1a induction by LPS in hepatocytes. **(A,B)** Primary hepatocytes isolated from WT and BRG1 LKO mice were infected with adenovirus carrying either TLR4 (Ad-TLR4) or GFP (Ad-GFP) followed by treatment with LPS (1 μg/ml) for 12 h. SREBP1a expression was examined by qPCR and Western. **(C,D)** HepG2 cells were transfected with siRNA targeting BRG1 or scrambled siRNA (SCR) followed by infection with Ad-GFP-TLR4 or Ad-GFP and treatment with LPS (1 μg/ml) for 12 h and SREBP1a expression was examined by qPCR and Western.

Having demonstrated that BRG1 may regulate SREBP1a expression to mediate LPS-induced liver injury by directly activating TLR4 transcription, we asked whether TLR4 over-expression could overcome BRG1 deficiency to restore SREBP1a expression. Adenovirus carrying either TLR4 expression vector (Ad-TLR4) or a control vector (Ad-GFP) was used to infect primary hepatocytes isolated from the BRG1 LKO mice. As shown in Figures 4A,B, Ad-TLR4 infection more than compensated the reduction of TLR4 expression in the LKO cells and brought the levels of SREBP1a expression to those observed in WT cells. Similarly, Ad-TLR4 infection in HepG2 cells offset the crippling effect of BRG1 depletion by maintaining SREBP1a expression (Figures 4C,D).

### TLR4 Over-Expression or SREBP1a Over-Expression Restores Liver Injury in BRG1 Deficient Mice

**FIGURE 5:**
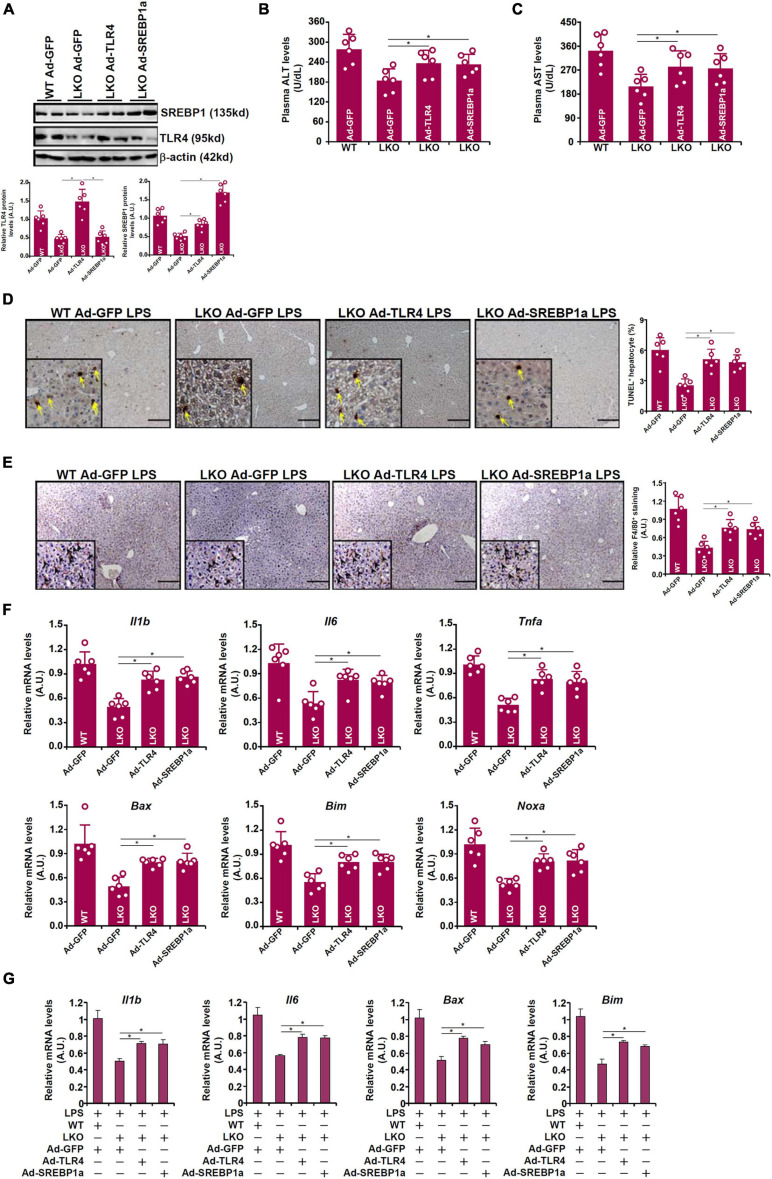
TLR4 over-expression or SREBP1a over-expression restores liver injury in BRG1 deficient mice. **(A–F)** WT and BRG1 LKO mice were injected via tail veinadenovirus carrying either TLR4 (Ad-GFP-TLR4), SREBP1a (Ad-GFP-SREBP1a), or GFP (Ad-GFP). 2 week later, the mice were injected with injected peritoneally with LPS (15 mg/kg) and sacrificed 24 h after the injection. Gene expression levels were examined by Western **(A)**. Plasma ALT levels **(B)**. Plasma AST levels **(C)**. Paraffin sections were stained with F4/80 **(D)**. Paraffin sections were stained with TUNEL **(E)**. Gene expression levels were examined by qPCR **(F)**. *N* = 6 mice for all groups. **(G)** Primary hepatocytes were isolated from WT or BRG1 LKO mice and transduced with indicated andenoviral particles. The cells were with LPS (1 μg/ml) for 12 h and gene expression was examined by qPCR.

Because attenuation of LPS-induced liver injury in the BRG1 LKO mice could be attributed to TLR4/SREBP1a down-regulation, we tested the hypothesis that re-introduction of exogenous TLR4 or SREBP1a might enable LPS to induce the same magnitude of liver injury in these mice as opposed to the WT mice. Adenovirus carrying TLR4 expression vector or SREBP1a vector or control vector was injected into the mice via tail vein followed by LPS injection. As shown in Figure 5A, Ad-TLR4 infection restored the expression of both TLR4 and SREBP1a whereas Ad-SREBP1a infection restored SREBP1a expression without altering TLR4 expression in the LKO livers. Exogenous TLR4 or SREBP1a overcame the BRG1 deficiency in the LKO livers by restoring LPS-induced liver injury as shown by plasma ALT (Figure 5B) and AST (Figure 5C) levels, by TUNEL staining (Figure 5D), by F4/80 staining (Figure 5E), and by qPCR measurements of pro-inflammatory and pro-apoptotic gene expression (Figure 5F). When primary hepatocytes were freshly isolated from WT or LKO mice and transduced with adenovirus carrying TLR4 expression vector or SREBP1a vector or control vector followed by LPS treatment, it was observed that both TLR4 over-expression and SREBP1a over-expression were able to partially overcome BRG1 deficiency and normalize the levels of pro-inflammatory mediators and pro-apoptotic factors (Figure 5G).

### Correlation of BRG1 and TLR4 in Human Liver Biopsy Specimens

**FIGURE 6:**
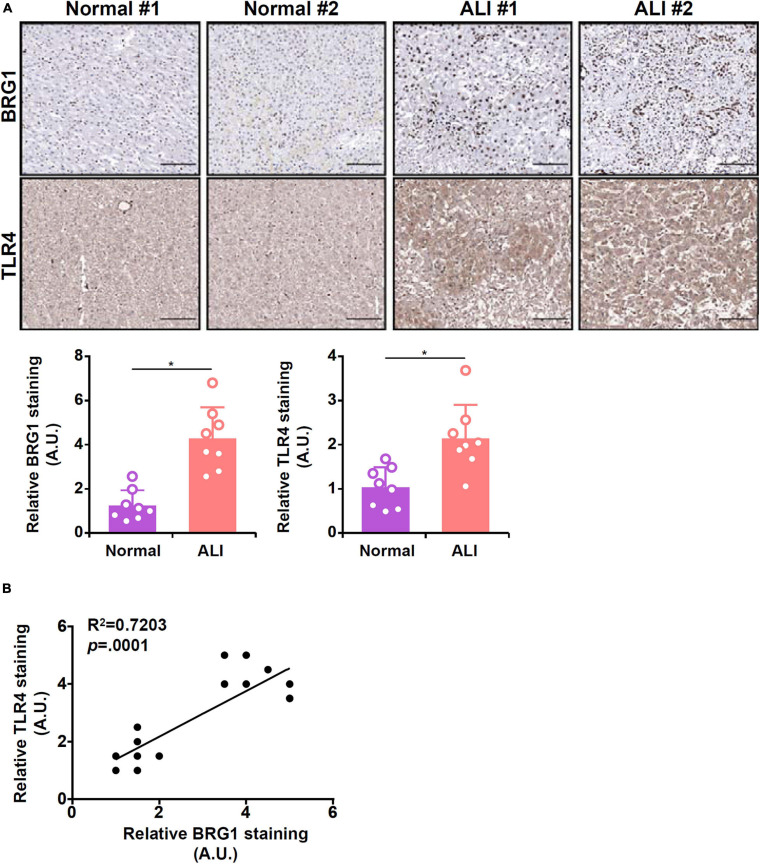
Expression levels of BRG1 and TLR4 in human liver biopsy specimens. **(A)** Representative images of BRG1 and TLR4 staining in liver biopsy specimens of patients diagnosed with acute liver injury (ALI). **(B)** Linear regression was performed with Graphpad Prism.

Finally, we probed the relationship between BRG1 and TLR4 expression in human liver biopsy specimens. As shown in Figure 6A, immunohistochemical staining revealed that both BRG1 levels and TLR4 levels were relatively low in the normal liver specimens but were markedly elevated in the specimens of ALI. Regression analysis showed a positive correlation between BRG1 and TLR4 expression (Figure 6B).

## Discussion

Septic shock represents a major cause for admissions into the intensive care unit (ICU) and the leading cause of deaths in non-coronary ICUs (Martin et al., 2003; Singer et al., 2016). Although septic shock causes the dysfunction of multiple organs, mortality rates of patients with liver failure tend to be the highest (Waseem et al., 2018). Therefore, effective interventional strategies that ameliorate liver dysfunction serve to boost the overall survival of patients with septic shock. Here we describe a novel transcriptional mechanism whereby the chromatin remodeling protein BRG1 contributes to liver injury in a mouse model of LPS-induced septic shock.

It has been previously shown that SREBP1a mediates the hepatic inflammatory response during septic shock (Im et al., 2011). Our data suggest that BRG1 deficiency leads to down-regulation of SREBP1a indirectly via TLR4. Because TLR4 is positioned at the very top of LPS-provoked signaling, there certainly are other possibilities that should be considered when interpreting these data. Liu et al. have found a global decrease in NF-κB activity in the TLR4-null hepatocytes compared to WT hepatocytes treated with LPS (Liu et al., 2002). BRG1 is a well-established co-factor for NF-κB (Fang et al., 2013; Zhang et al., 2019). Therefore it is possible that BRG1 may regulate liver injury by modulating NF-κB activity in hepatocytes. In addition, BRG1 is directly involved in the transcription of pro-inflammatory genes (Ramirez-Carrozzi et al., 2006) and pro-apoptotic genes (Napolitano et al., 2007). We have previously shown that BRG1 can interact with SREBP and facilitate SREBP-dependent transcriptional events related to lipogenesis and cholesterogenesis in hepatocytes (Li et al., 2018b; Fan et al., 2020). SREBP1 has been shown to directly bind to the promoter regions of pro-inflammatory genes (Oishi et al., 2017) and pro-apoptotic genes (Wang et al., 2005; Gibot et al., 2009), raising the intriguing possibility that BRG1 may co-occupy the gene promoters with SREBP1 and potentiate the transcriptional activity of SREPB1 to stimulate cellular inflammation and/or apoptosis. Finally, SREBP1 activity is not only determined by its overall expression but its liberation from the membrane and translocation into the nucleus (Horton et al., 2002). Diomede et al. have observed that LPS administration in mice promotes hepatic SREBP1 maturation in a TLR4-dependent manner (Diomede et al., 2001). Whether BRG1 can contribute to this process remains to be determined.

We show here that BRG1 is recruited to the *Tlr4* promoter region containing a conserved hypoxia response element (HRE). Previously we have reported that BRG1 can interact with HIF-1α and mediate HIF-1α induced transcription of IL-33 gene (Liu et al., 2019b), MRP8 gene (Li et al., 2020c), and KDM3A gene (Li et al., 2019d) respectively. These data appear to point to an interesting scenario wherein a HIF-1α-BRG1 complex regulates the hepatic transcriptome to promote liver dysfunction. Indeed, mice with hepatocyte-specific HIF-1α deletion exhibit similar phenotypes as the BRG1 LKO mice in a number of different animal models of liver injury. For instance, the HIF-1α LKO mice are resistant to LPS-induced liver injury (Nath et al., 2011). Hepatocyte-specific HIF-1α deficiency also protects the mice from obesity and steatosis (Lee et al., 2019) and liver necrosis caused by hepatotoxic substances (Sparkenbaugh et al., 2011; Roychowdhury et al., 2014). The genomewide mechanistic insights underlying the functional overlapping between HIF-1α and BRG1 are currently lacking. There are several reports demonstrating genomewide binding patterns of HIF-1α (Schodel et al., 2011; Smythies et al., 2019) and BRG1 (Bossen et al., 2015; Barutcu et al., 2016; Raab et al., 2017) using ChIP-seq; none of these studies examined (primary) hepatocytes. Future studies should focus on deciphering whether the functional overlapping between HIF-1α and BRG1 can be explained by shared transcriptional programs.

There are several major limitations regarding the approaches and conclusions of the present study. First, a string of recent studies have claimed that BRG1 possesses a protective role in hepatic ischemia-reperfusion injury (Ge et al., 2017a, b, c). The mechanism, accordingly to Ge et al., lies in the observation that BRG1 can interact with the anti-oxidative transcription factor Nrf2 to activate HO-1 transcription in hepatocytes, which may not be the rate-limiting process in LPS-induced liver injury as investigated in the present study. Alternatively, whereas a hepatocyte-specific BRG1 deletion mouse strain was used in the present study, Ge et al. in their series of investigations used a mouse strain harboring systemic BRG1 over-expression, which may engender non-hepatocyte autonomous effects. Second, a single mouse model (LPS injection) was harnessed to assess the role of BRG1 in ALI. Other models widely exploited by the field to study ALI include dual injection of LPS plus pyrazole (a CYP2E1 inducer), dual injection of LPS plus D-galactosamine, and the cecal ligation and puncture (CLP) procedure (Louis et al., 1997; Lu et al., 2005; Zhu et al., 2013). It would be of great help to ascertain the role of BRG1 in additional mouse models. Third, no transcriptomic analysis (e.g., RNA-seq) was performed to screen for genomewide targets downstream of BRG1 that might contribute to the observed phenotype. Wang et al. (2019) have compared the transcriptomes of WT livers and BRG1-null livers following partial hepatectomy in mice by RNA-seq and found that the p53 pathway is preferentially activated by BRG1 deficiency. Because several studies have demonstrated a protective role for p53 in acute organ injuries (Liu et al., 2009; Sun et al., 2018), it is tempting to speculate that attenuation of liver injury by BRG1 deficiency might be attributed to a secondary up-regulation of p53. These issues certainly deserve further attention.

In summary, our data add another layer of evidence that supports BRG1 as a common mediator of liver injury. Small-molecule BRG1 inhibitors have been designed and proven effective in the intervention of malignant cancers both in cell culture (Wu et al., 2016) and in animal models (Ding et al., 2019). It would be of great interest to determine whether these compounds can be considered as a reasonable approach to treat liver disorders.

## Data Availability Statement

The original contributions presented in the study are included in the article/supplementary material, further inquiries can be directed to the corresponding author/s.

## Ethics Statement

The animal study was reviewed and approved by Nanjing Medical University Ethics Committee on Humane Treatment of Experimental Animals.

## Author Contributions

XF and ZZ conceived the project. WD, YwZ, YxZ, and ZF designed experiments, performed experiments, and collected, analyzed the data. XF and ZZ provided funding. YX wrote the manuscript. All authors contributed to the article and approved the submitted version.

## Conflict of Interest

The authors declare that the research was conducted in the absence of any commercial or financial relationships that could be construed as a potential conflict of interest.
